# Fabrication of Micro-Holes with High Aspect Ratios in C_f_/SiC Composites Using Coaxial Waterjet-Assisted Nanosecond Laser Drilling

**DOI:** 10.3390/mi16070811

**Published:** 2025-07-14

**Authors:** Chenhu Yuan, Zenggan Bian, Yue Cao, Yinan Xiao, Bin Wang, Jianting Guo, Liyuan Sheng

**Affiliations:** 1 Department of Mechanical and Automotive Engineering, South China University of Technology, Guangzhou 510641, China; 202310180121@mail.scut.edu.cn; 2 PKU-HKUST Shenzhen-Hong Kong Institution, Shenzhen 518057, China; b1127555895@163.com (Z.B.); caoyue@nimte.ac.cn (Y.C.); ynxiao_pkusz@yeah.net (Y.X.); jiantingguo@yeah.net (J.G.); 3 Shenzhen Institute, Peking University, Shenzhen 518057, China; 4 Ningbo Institute of Materials Technology and Engineering, Chinese Academy of Sciences, Ningbo 315201, China; 5 Institute of Metal Research, Chinese Academy of Sciences, Shenyang 110016, China

**Keywords:** coaxial waterjet-assisted nanosecond laser drilling, C_f_/SiC composites, ablation mechanism, waterjet velocity, micro-holes with high aspect ratios

## Abstract

In the present study, the coaxial waterjet-assisted nanosecond laser drilling of micro-holes in C_f_/SiC composites, coupled with nanosecond laser drilling in air for fabricating micro-holes with high aspect ratios, were investigated. The surface morphology, reaction products, and micro-hole shapes were thoroughly examined. The results reveal that, for the coaxial waterjet-assisted nanosecond laser drilling of micro-holes in the C_f_/SiC composite, the increasing of waterjet velocity enhances the material removal rate and micro-hole depth, but reduces the micro-hole diameter and taper angle. The coaxial waterjet isolates the laser-ablated region and cools down the corresponding region rapidly, leading to the formation of a mixture of SiC, SiO_2_, and Si on the surface. As the coaxial waterjet velocity increases, the morphology of residual surface products changes from a net-like structure to individual spheres. Coaxial waterjet-assisted nanosecond laser drilling, with a waterjet velocity of 9.61 m/s, achieves micro-holes with a good balance between efficiency and quality. For the fabrication of micro-holes with a high aspect ratio in C_f_/SiC composites, micro-holes fabricated by nanosecond laser drilling in air exhibit obvious taper features, which should be ascribed to the combined effects of spattering slag, plasma, and energy dissipation. The application of coaxial waterjet-assisted nanosecond laser drilling on micro-holes fabricated by laser drilling in air effectively expands the hole diameter. The fabricated micro-holes have very small taper angles, with clean wall surfaces and almost no reaction products. This approach, combining nanosecond laser drilling in air followed by coaxial waterjet-assisted nanosecond laser drilling, offers a promising technique for fabricating high-quality micro-holes with high aspect ratios in C_f_/SiC composites.

## 1. Introduction

With the rapid advancement of the aerospace industry, the demand for higher thrust in aero-engines has significantly increased, emphasizing the importance of improving the thrust-to-weight ratio [1]. To achieve this goal, a variety of emerging materials have been developed and applied as hot-end components in the aerospace industry [2,3,4]. Ceramic matrix composites (CMCs) have gained considerable attention due to their exceptional high-temperature performance. Among the more mature CMCs, the C_f_/SiC composite (carbon fiber reinforced silicon carbide matrix) exhibits a range of remarkable properties, including low density, excellent thermal stability, superior toughness, and high specific strength and modulus, making it an ideal material for high-temperature applications [5,6]. Currently, the C_f_/SiC composite is used in the critical hot-end components of aero-engines, turbines, rocket engine combustion chambers, and other essential systems [7]. Due to its high melting point and inherent oxidation resistance, the C_f_/SiC composite can withstand higher temperatures while significantly reducing weight, thereby enhancing the thrust-to-weight ratio. As a critical component in aero-engines, the C_f_/SiC composite must maintain reliable performance in harsh environments over extended periods, necessitating air cooling for these components [8,9]. Unlike superalloys, the high stiffness and intrinsic brittleness of SiC make machining this composite using conventional methods particularly challenging [10].

Generally, conventional machining techniques, such as drilling, grinding, and cutting, are effective for processing homogeneous materials with acceptable ductility, allowing for high-precision processing; however, for heterogeneous CMCs with high stiffness or brittleness, the high, yet unstable, forces exerted by machine tools can lead to defects such as cracks, stripping, or fiber pull-out. This issue is particularly pronounced in C_f_/SiC composites, where the disparity in physical properties between the carbon fibers and the SiC matrix exacerbates these defects [11]. As a result, surface defects such as burrs, tearing, edge collapse, and delamination can appear on the processed C_f_/SiC composite, compromising its service performance [12]. Additionally, the C_f_/SiC composites can cause high wear rates on machine tools, which negatively impacts the consistency of machining quality. Therefore, conventional machining methods with contacting modes are not an optimal choice for processing C_f_/SiC composites, especially when drilling small-sized shapes. Wang et al. [13] investigated a novel composite stepped cone diamond bit for the ultrasonic machining of CMCs, which reduced the tearing size by an average of 30%. Furthermore, this new tool exhibited reduced dependence on tearing size and processing variables compared to conventional drill bits, significantly enhancing the quality of the drilled outlets. For micro-holes with high aspect ratios, however, the fluidity of abrasive particles becomes restricted, reducing processing efficiency greatly [14,15]. Electrical discharge machining (EDM) can be used to machine micro-holes in C_f_/SiC composites, but its processing quality is heavily influenced by the electrical conductivity of composites [16]. Due to the porosity of C_f_/SiC composites, their electrical conductivity is low, which undermines both processing continuity and surface quality. Moreover, the limited flow of cooling liquid in micro-holes further decreases surface quality. As a result, achieving high-quality micro-holes via micro-machining with specific solid media proves challenging. A fully non-contact machining technique is expected to provide better processing quality.

Compared to conventional machining methods, high-energy laser processing offers several advantages, including non-contact operation, high beam power density, controllable energy concentration, no material limitation, good adaptability to complex 3D surfaces, and high processing efficiency and precision. These benefits contribute to the high-quality processing of small-sized structures and reduce defect formation [17,18,19]. As a result, an increasing number of researchers are focusing on investigating laser processing techniques for C_f_/SiC composites. Especially for micro-holes, the high precision and minimal damage of laser drilling enable effective processing of C_f_/SiC composite components, such as combustion chambers and heat insulation tiles in aero-engines. Zhang et al. [20] used continuous wave lasers to prepare micro-holes in C_f_/SiC composites, revealing that the ablation mechanism of SiC and carbon fiber follows a pattern similar to “onion peeling”. They also suggested that medium-output laser power can achieve better surface morphology during processing. Wu et al. [21] machined C_f_/SiC composites with a unidirectional carbon fiber orientation and demonstrated the varying surface morphology caused by the carbon fibers. Liu et al. [22] investigated the drilling of micro-holes in 2.5D C_f_/SiC composites using millisecond lasers, revealing different ablation evolution behaviors in regions with varying carbon fiber orientations. Previous studies [23,24,25] on the picosecond laser processing of C_f_/SiC composites showed that laser energy, feed rate, and scanning mode significantly influenced the quality of drilled micro-holes, as evidenced by the presence of pores, spattering, and debris. Specifically, the spiral scanning mode, combined with appropriate laser energy and feed rate, could achieve micro-holes with large aspect ratios. The laser machining of C_f_/SiC composites in air, however, leads to significant oxidation and spattering, and the subsequent removal of oxides and spattering from the processed surface, especially on the walls of micro-holes, remains a critical challenge.

Compared to simple laser drilling in air, medium-assisted laser drilling can achieve better surface quality for micro-holes in C_f_/SiC composites. Zhai et al. [26] applied argon shielding gas in the femtosecond laser processing of C_f_/SiC composites and conducted theoretical calculations, which demonstrated the effective inhibition of edge oxidation in the processed region. Cao et al. [27] investigated various gas-assisted nanosecond laser drilling techniques and found that inert gases improved the processed surface quality but decreased ablation efficiency, while reactive gases increased ablation efficiency but compromised surface quality. This phenomenon was attributed to enhanced oxidation and material removal by gas flow. Nevertheless, laser drilling of CMCs in air resulted in a noticeable heat-affected zone (HAZ), which influenced their service life and performance. Wang et al. [28] demonstrated that the mechanical erosion caused by supersonic gas flow increased the laser ablation efficiency and stabilized the ablated surface compared to a static air environment. Recent research on the water-assisted laser processing of CMCs showed that water effectively shielded the laser-ablated area and eliminated oxidation [29,30]. Additionally, the high thermal capacity of water helped effectively remove dissipated heat, thereby restraining the extension of the heat-affected zone (HAZ); however, fully cooling the area with assisted water could reduce subsequent laser ablation efficiency. Furthermore, the initial ultrafine micro-holes could also affect the flow of water inside. A combined process, using low-pressure waterjet-assisted picosecond laser drilling followed by post-electrochemical machining, was shown to effectively remove slag attachment and recast layers, optimize hole geometry, and achieve high surface quality, demonstrating strong potential for the high-precision fabrication of film cooling holes [31]. Therefore, it is essential to develop a process that combines the advantages of both water-assisted and gas-assisted laser drilling.

Based on previous research on the laser processing of C_f_/SiC composites, it is evident that most studies primarily focus on single laser drilling technology, while research on water-assisted laser drilling methods remains limited. Therefore, in this study, nanosecond laser drilling in air and coaxial waterjet-assisted nanosecond laser drilling were combined to fabricate micro-holes with a high aspect ratio in C_f_/SiC composites. Given the limited research on waterjet-assisted laser drilling, this was the main focus of the present study, which specifically explores the effect of the main factor, waterjet velocity. The optimal parameters were applied to process the pre-drilled micro-holes using nanosecond laser drilling in air. The shape of the micro-holes, surface morphology, and reaction products were systematically characterized. The goal was to develop a more effective and high-quality laser machining procedure for C_f_/SiC composites.

## 2. Materials and Experiments

### 2.1. C_f_/SiC Composite

**Figure 1:**
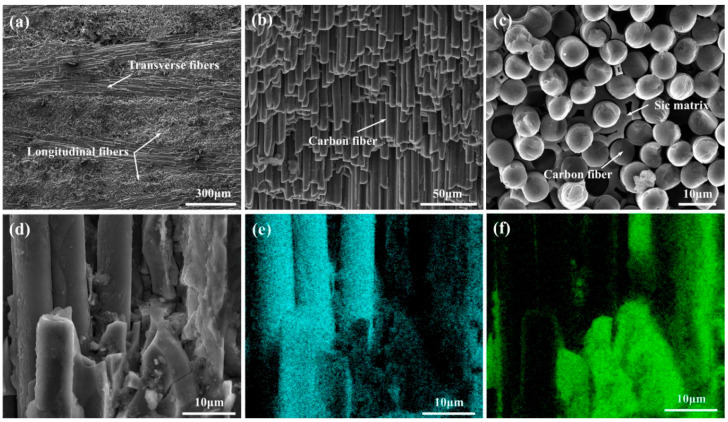
Morphology of the as-received C_f_/SiC composite: (**a**) Cross-sectional microstructure, (**b**) Arrangement of carbon fibers, (**c**) Morphology of carbon fibers, (**d**) Interface of carbon fiber and SiC matrix, (**e**) Distribution of C in image (**d**), (**f**) Distribution of Si in image (**d**).

In the present study, the C_f_/SiC composites used were purchased from the Shanghai Institute of Ceramics, Chinese Academy of Sciences, with dimensions of 50 mm × 50 mm × 5 mm. These composites were fabricated using the precursor impregnation pyrolysis (PIP) technique and were primarily composed of an SiC matrix, carbon fibers, and a pyrolytic carbon interface layer (Pyc) [32]. The as-received C_f_/SiC composite featured a 2D stitched structure, where the carbon fibers, embedded in SiC, were arranged regularly, as shown in Figure 1. The thickness of the single carbon fiber layer was measured to be 258.5 ± 16.5 μm, with the carbon fibers densely arranged, as shown in Figure 1a,b. The carbon fibers exhibited various gaps, with distances to the order of several micrometers, as shown in Figure 1c. In different layers, the carbon fiber bundles intersected at a 90° angle. Further observations of the interface between the carbon fibers and the SiC matrix revealed insufficient bonding, as the SiC could easily debond from the carbon fiber surface, as shown in Figure 1d. The analyses of elemental distributions also verified this deduction, as there was almost no SiC residual on the carbon fiber surface, as shown in Figure 1e,f. The carbon fibers, serving as the reinforcement in the C_f_/SiC composite, occupied nearly half of the composite’s volume, although small voids were present. The detailed physical properties of the C_f_/SiC composite are presented in Table 1. Prior to laser drilling, the C_f_/SiC composite samples were cleaned using ultrasonic-assisted anhydrous ethanol for 20 min.

### 2.2. Laser Drilling Equipment and Parameters

**Figure 2:**
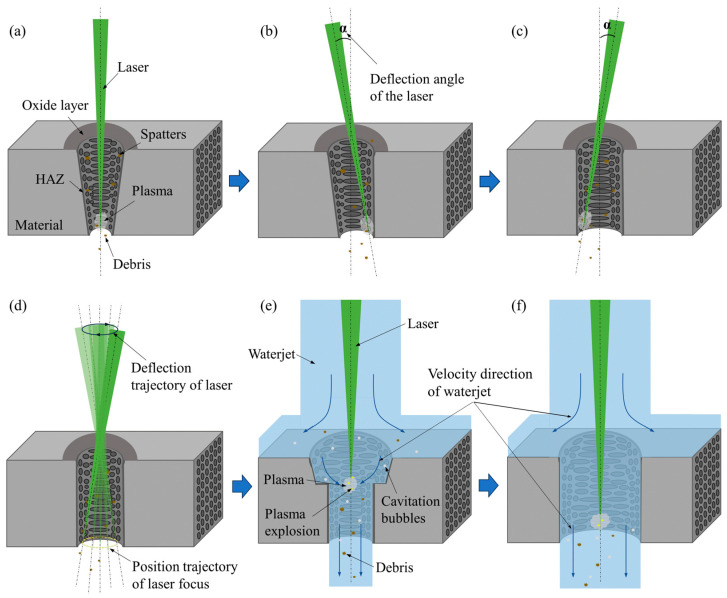
Schematic diagram of the laser-drilling process of C_f_/SiC composites: (**a**) Nanosecond laser drilling in air for initial micro-holes, (**b**–**d**) Nanosecond laser drilling in air with reflection of laser beam angle decreasing taper angle of micro-holes, (**e**,**f**) Expanding micro-holes by coaxial waterjet-assisted nanosecond laser drilling.

The primary objective of the present research is to drill micro-holes with diameters ranging from 400 μm to 600 μm in C_f_/SiC composites. To achieve this, the laser drilling process consists of two steps: nanosecond laser drilling in air and coaxial waterjet-assisted nanosecond laser drilling, as shown in Figure 2. The nanosecond laser drilling in air involved initial micro-holes created by direct laser ablation, followed by a reduction in the micro-holes’ taper angle through laser beam deflection, as depicted in Figure 2a–d. Given that the laser drilling of C_f_/SiC composites in air with a deflected laser beam angle had been previously explored in our research [33], the optimized parameters were directly applied to prepare the pre-drilled micro-holes for this study. As a result, the experimental outcomes for laser drilling in air are not included here. The focus of the current research is on the coaxial waterjet-assisted nanosecond laser drilling, particularly on the effect of waterjet velocity.

**Figure 3:**
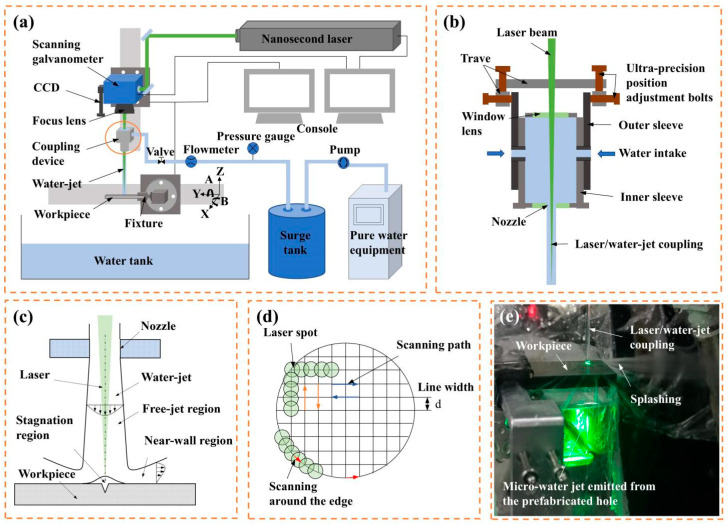
Schematic diagram of the coaxial waterjet-assisted nanosecond laser drilling of C_f_/SiC composite: (**a**) Whole coaxial waterjet-assisted laser drilling system, (**b**) Detailed structure of waterjet coupling system, (**c**) Evolution of waterjet with distance and laser coupling, (**d**) Scanning route of laser beam during drilling, (**e**) Drilled C_f_/SiC composite in designed laser processing platform.

The schematic diagram of the coaxial waterjet-assisted nanosecond laser drilling is shown in Figure 3a. The coaxial waterjet-assisted nanosecond laser drilling system primarily consists of a nanosecond laser, optical transmission system, light-water coupling system, five-axis moving platform, CCD, and a pure water pressurization system [30]. A nanosecond laser (Edge Wave InnoSlab IS8I-E) with a wavelength of 532 nm was used as the light source. To achieve waterjet coupling, a specialized system was designed (its structure is illustrated in Figure 3b). The water inlet was symmetrically placed on the sides of the water chamber to balance the pressure, while the water pressure was controlled by adjusting the pump voltage, which in turn regulated the waterjet velocity. Unlike traditional water-guided laser systems, where energy is transmitted through total reflection of the laser beam, the coaxial waterjet-assisted nanosecond laser drilling system focused the laser spot within the waterjet, facilitating the drilling of C_f_/SiC composites, as shown in Figure 3c. The focused laser spot moved precisely along a fixed path with the waterjet, removing the composite layer by layer. The laser spot scanning path and the filling scanning path were designed as shown in Figure 3d. The C_f_/SiC composites were drilled by the designed laser processing platform, as shown in Figure 3e. The detailed parameters for the coaxial waterjet-assisted nanosecond laser drilling processing of C_f_/SiC composites were preset, as listed in Table 2. Based on theoretical analysis and the experimental verification of stable laminar flow in the processing zone, the waterjet velocities were selected as 4.68 m/s, 6.48 m/s, 8.16 m/s, 9.61 m/s, and 11.00 m/s.

### 2.3. Microstructure and Surface Characterization

After laser drilling, the processed C_f_/SiC composite samples were cut using a diamond-cutting machine, then cleaned in ethanol with ultrasonic assistance for 20 min to remove residues. The composite samples were subsequently characterized to examine the influence of waterjet velocity and the combined laser drilling process. A laser confocal scanning microscope (LCSM, Keyence VX-200, Keyence Co., Ltd., Osaka Prefecture, Japan) was used to analyze the surface morphology, depth, and diameter of the micro-holes. The microstructure and elemental distribution of the processed composite surface were examined using a scanning electron microscope (SEM, FEI Quanta FEG 250, Hillsboro, OR, USA). The phase constituents of the composite samples were analyzed using an X-ray diffractometer (XRD, D8 ADVANCE, Karlsruhe, Germany). Additionally, X-ray photoelectron spectroscopy (XPS, AXIS SUPRA, Manchester, UK) was employed to examine the changes in chemical bonds within the laser-ablated regions under different assisting environments.

## 3. Results and Discussion

### 3.1. Effect of Waterjet Velocity on Coaxial Waterjet-Assisted Nanosecond Laser Drilling of C_f_/SiC Composite

**Figure 4:**
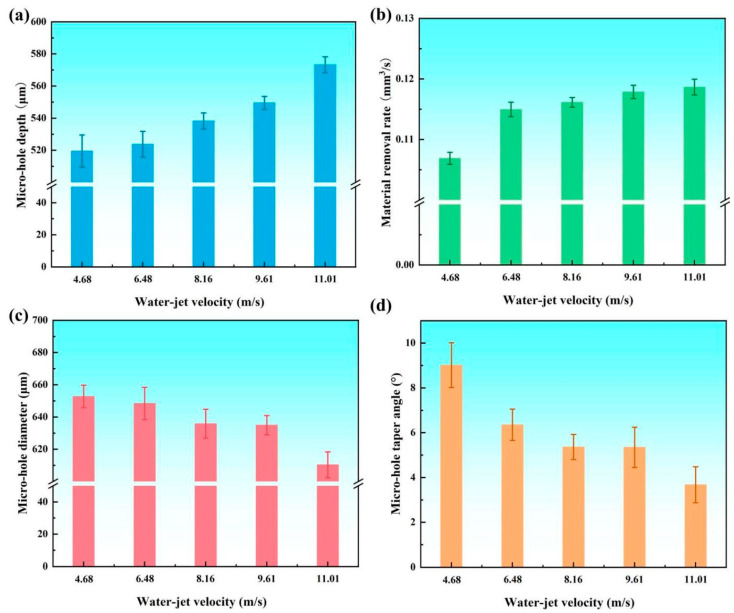
Variation of coaxial waterjet-assisted nanosecond laser-drilled micro-holes in C_f_/SiC composite at different waterjet velocities: (**a**) Micro-hole depth, (**b**) Material removal rate, (**c**) Micro-hole entrance diameter, (**d**) Micro-hole taper (waterjet velocity varied as 4.68 m/s, 6.48 m/s, 8.16 m/s, 9.61 m/s, 11.00 m/s).

In waterjet-assisted laser drilling, waterjet velocity plays a crucial role in removing dissipated laser energy and influencing the condition of the laser-ablated area [34]. To investigate the effect of waterjet velocity, the nanosecond laser drilling parameters were preset based on previous research [10,30]. The fixed nanosecond laser drilling parameters were as follows: a laser repetition rate of 30 kHz, a single pulse energy of 0.65 mJ, a defocus distance of 0 mm, a laser spot overlap rate of 80%, and a processing time of 5 s. The waterjet velocity was varied as the values set in experimental. Based on LCSM analyses, the diameter, depth, taper angle, and material removal rate (MRR) of micro-holes were investigated, as shown in Figure 4. As the waterjet velocity increases, the micro-holes depth gradually increases from 519 µm to 573 µm, as shown in Figure 4a. Notably, the rate of increase in micro-hole depth is more pronounced at higher waterjet velocities compared to lower waterjet velocities, indicating that a higher waterjet velocity significantly contributes to greater micro-hole depth. The MRR also shows an upward trend with increasing waterjet velocity, rising from 0.107 mm^3^/s to 0.118 mm^3^/s, as shown in Figure 4b. This improvement can be attributed to the enhanced removal of molten material and debris by the waterjet, which facilitates continuous laser ablation and prevents redeposition on the hole walls. In addition, the dynamic water flow suppresses plasma-shielding effects and clears vaporized particles from the processing zone, allowing more laser energy to couple with the material surface, improving ablation efficiency. When the waterjet velocity exceeds 6.48 m/s, however, the rate of increase in MRR slows. This reduction in growth rate is likely due to the intensified cooling effect at higher waterjet velocities, which accelerates heat dissipation in the ablation zone and lowers the local temperature, thereby reducing the thermal energy available for material removal. Furthermore, excessive water flow may destabilize the laser-induced plasma plume and scatter part of the incident laser beam, diminishing the overall ablation efficiency. In contrast, the micro-holes’ entrance diameter decreases with increasing waterjet velocity, from 652 µm to 615 µm, as shown in Figure 4c. This suggests that the increased MRR associated with higher waterjet velocity primarily contributes to an increase in micro-hole depth. Furthermore, the micro-holes’ taper angle decreases as waterjet velocity increases, as shown in Figure 4d. This phenomenon implies that a higher waterjet velocity helps improve both the shape and depth of the micro-holes.

**Figure 5:**
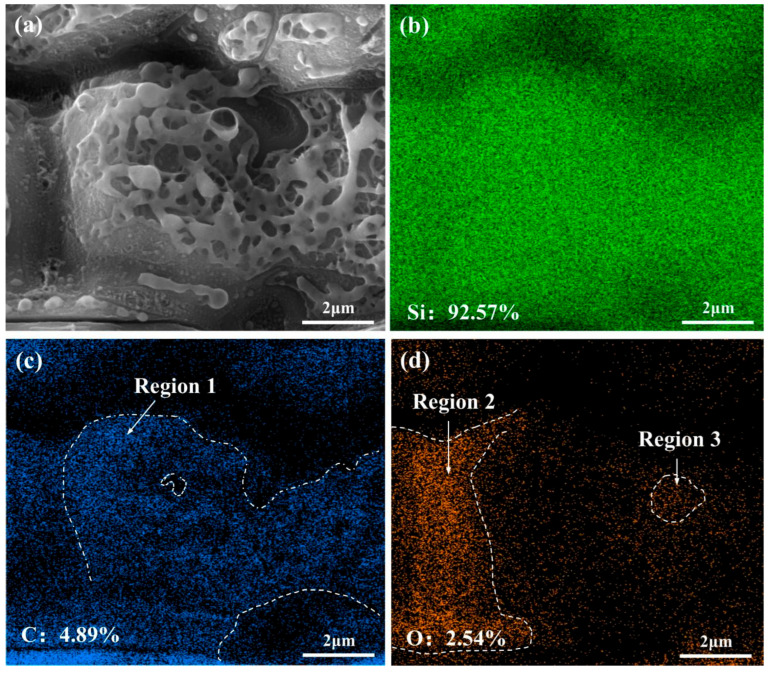
The SEM observation and EDS analyses on the coaxial waterjet-assisted nanosecond micro-hole wall at a waterjet velocity of 4.68 m/s: (**a**) Morphology of products, (**b**) Distribution of Si, (**c**) Distribution of C, (**d**) Distribution of O (elemental content is atomic percent).

To investigate the effect of waterjet velocity, the SEM observations were conducted on the internal surface of micro-holes, and the results are shown in Figure 5. The typical SEM image of the coaxial waterjet-assisted nanosecond laser-drilled C_f_/SiC composite surface, with a waterjet velocity of 4.68 m/s, reveals two kinds of reaction products, as shown in Figure 5a. The big products form the net-like morphology, while the small ones exhibit a daisy pistil-like shape and are homogeneously distributed on the matrix surface. The elemental distribution analyses of these reaction products reveal that the net-like reaction products are primarily composed of Si with some C, while the daisy pistil-like products mainly contain Si and some O, as shown in Figure 5b–d. These observations suggest that a decomposition reaction occurs under near-vacuum conditions, resulting in the breakdown of SiC and minor oxidation [30,35]. The EDS results further exhibit that the Si, C, and O content in the observed area is 92.57%, 4.89%, and 2.54%, respectively. Closer examination of the surface shows that the net-like reaction products in region 1 have little O but relatively more C content, indicating that they are composed mainly of Si and SiC. In contrast, region 2, where the daisy pistil-like products are found, is primarily composed of Si and O, suggesting the formation of SiO_2_ on the matrix surface. According to previous studies [10,36], high-density laser irradiation leads to the vaporization of C_f_/SiC composite and subsequent decomposition. With the protection of the waterjet, the laser-irradiated small region is mostly shielded from the oxidation, which significantly restrains oxidation and promotes the deposition of the Si and SiC phases.

**Figure 6:**
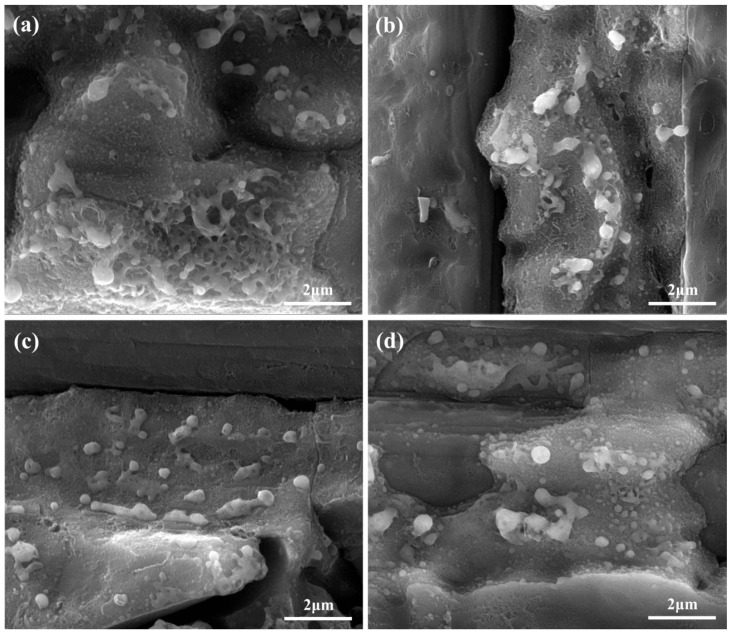
The typical SEM observations of the coaxial waterjet-assisted nanosecond laser-drilled micro-hole wall morphology at different waterjet velocities: (**a**) 6.48 m/s, (**b**) 8.16 m/s, (**c**) 9.61 m/s, (**d**) 11.01 m/s.

To further study the effect of waterjet velocity, the surfaces of coaxial waterjet-assisted nanosecond laser-drilled C_f_/SiC composites with increased waterjet velocity were observed by SEM. As shown in Figure 6, all laser-processed composite surfaces contain two types of reaction products, i.e., big one and small ones, which are consistent with the laser-processed composite surface at a waterjet velocity of 4.68 m/s. The quantity of big reaction products decreases, however, as the waterjet velocity increases, and their morphology changes from a net-like shape to individual spheres. At a waterjet velocity of 6.48 m/s, the big reaction products still exhibit the net-like shape, but their quantity slightly decreases, as shown in Figure 6a. When the waterjet velocity increases to 8.16 m/s, the big reaction products almost lose their net-like shape and become more individual, as shown in Figure 6b. For the composite processed at a waterjet velocity of 9.61 m/s, the big reaction products are spheroidized, as shown in Figure 6c. When the waterjet velocity increases to 11.01 m/s, the average dimension of big reaction products decreases, as shown in Figure 6d. It is interesting that the morphology of small reaction products changes little with the variation of waterjet velocity. That indicates that increased waterjet velocity could restrain the deposition of vaporized composite but exerts little influence on the oxidation.

**Figure 7:**
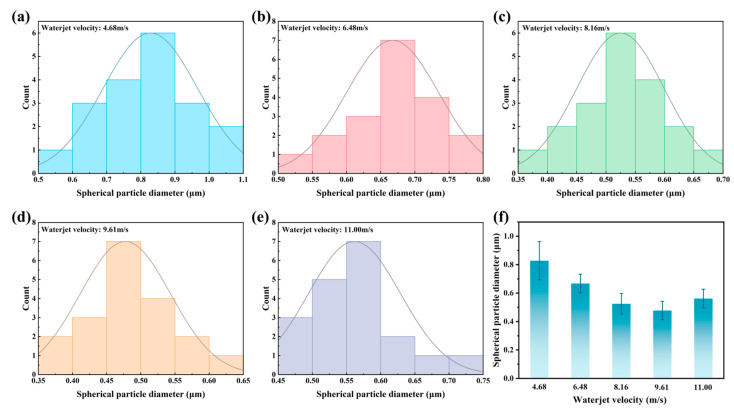
Distribution of spherical particle diameters at the bottom of micro-holes processed by coaxial waterjet-assisted nanosecond laser drilling at different waterjet velocities: (**a**) 4.68 m/s, (**b**) 6.48 m/s, (**c**) 8.16 m/s, (**d**) 9.61 m/s, (**e**) 11.01 m/s. (**f**) Summary of the overall average particle diameters.

The dimensions of the reaction products were statistically analyzed using the SEM image. With increasing waterjet velocity, the dominant particle size range in the histograms of spheroidized reaction products first decreases, then increases, as shown in Figure 7. The detailed statistical analysis shows that the diameters of the spherical particles formed on the composite surface processed at a waterjet velocity of 4.68 m/s mainly locate between 0.5 µm and 1.1 µm, as shown in Figure 7a. With the increasing of waterjet velocity, the diameter scopes of the spherical particles decrease gradually, as shown in Figure 7b–e. For the spherical particles formed on the composite surfaces processed at a waterjet velocity of 8.16 m/s, 9.61 m/s, and 11.01 m/s, their diameters mainly locate between 0.4 µm and 0.7 µm. The calculated average spherical particle diameter formed on the composite surfaces processed at a waterjet velocity of 4.68 m/s, 6.48 m/s, 8.16 m/s, 9.61 m/s, and 11.01 m/s are 0.83 µm, 0.67 µm, 0.52 µm, 0.46 µm, and 0.54 µm, respectively. The formed reaction products reach their minimum values at a waterjet velocity of about 9 m/s. These results suggest that there is an optimal jet velocity at which the removal of molten products and the suppression of redeposition are most effective, leading to finer and more uniform residual morphologies. Beyond this velocity, however, the intensified jet flow may encourage partial re-agglomeration or impede the complete detachment of vaporized materials, causing a slight increase in particle size.

**Figure 8:**
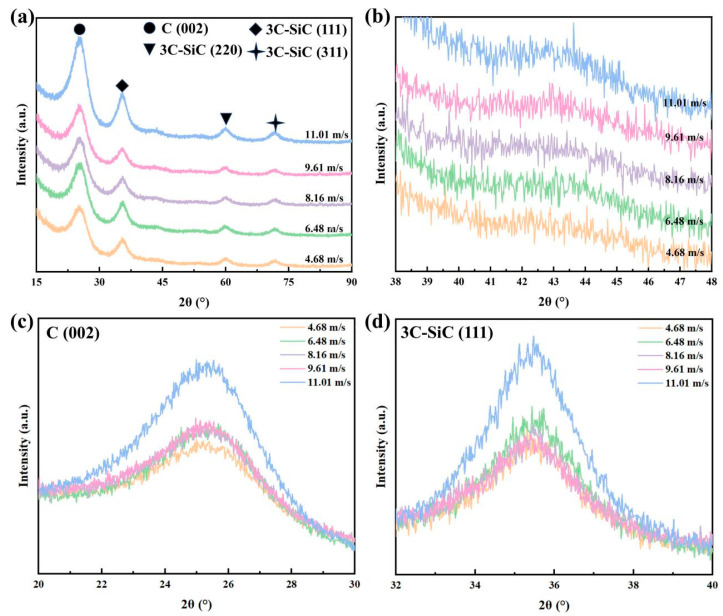
XRD patterns of the coaxial waterjet-assisted nanosecond laser-drilled C_f_/SiC composite under different waterjet velocities: (**a**) Full patterns of all specimens, (**b**) Enlarged XRD patterns between 40° and 46°, (**c**) Enlarged XRD patterns along 002_C_ diffraction peak, (**d**) Enlarged XRD patterns along 111_SiC_ diffraction peak.

The XRD analyses on the coaxial waterjet-assisted nanosecond laser-drilled C_f_/SiC composite were performed, and the results are shown in Figure 8. It is evident that the diffraction patterns mainly contain two phases of carbon and SiC, as shown in Figure 8a; however, the intensities of the diffraction peaks change with the increasing waterjet velocity. Specifically, the intensities of diffraction peaks for 002_C_ and 111_SiC_ initially increase, then slightly decrease, and finally show a significant increase. The increased intensities of these diffraction peaks suggest a reduction in the corresponding amorphous structure in the surface layer [30]. Moreover, the diffraction patterns between 40° and 46° display a broad feature, indicating the formation of an amorphous structure, as shown in Figure 8b. According to previous studies [37,38], a relatively weak broad diffraction peak corresponds to a combination of amorphous SiO_2_ and Si. The enlarged XRD patterns reveal in detail the phases of variation in the surface layer, as shown in Figure 8c,d. For the 002_C_ diffraction peak, increasing waterjet velocity results in a higher intensity of XRD patterns; however, the rate of increase is relatively small for C_f_/SiC composites processed at a waterjet velocity below 11.01 m/s. Similarly, for the 111_SiC_ diffraction peak, the intensity of the XRD pattern follows the same trend, with a noticeable increase starting at a waterjet velocity of 9.61 m/s, as shown in Figure 8d. The variation of XRD patterns further supports the above deduction that the waterjet could restrain the oxidation but cannot eliminate it. Increased waterjet velocity enhances the removal efficiency of vaporized composites and suppresses deposition, leading to a higher diffraction intensity.

**Figure 9:**
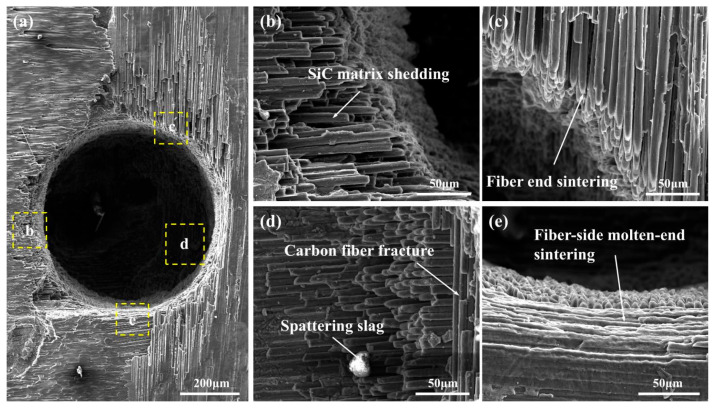
Typical SEM observations of the coaxial waterjet-assisted nanosecond laser-drilled micro-hole in the C_f_/SiC composite with a waterjet velocity of 9.61 m/s: (**a**) Morphology of laser-drilled micro-hole, (**b**) Morphology of carbon fibers partly covered with SiC, (**c**) Morphology of carbon fiber with typical ablated end, (**d**) Morphology of carbon fibers with axial direction perpendicular to micro-hole radial direction, (**e**) Morphology of micro-hole wall with sintering feature.

Based on the above analyses, it is evident that waterjet velocity has a diverse impact on the dimension and surface products of the coaxial waterjet-assisted nanosecond laser-drilled micro-holes in the C_f_/SiC composite. For the preset laser-drilling parameters, the micro-hole drilled at a waterjet velocity of 9.61 m/s exhibit a balanced performance. To further investigate the morphology features, the micro-holes drilled at a waterjet velocity of 9.61 m/s were chosen for analysis (their SEM observations are presented in Figure 9). The micro-holes exhibit a relatively clear and regular shape, as shown in Figure 9a; however, the laser processing has caused damage in the local area adjacent to the micro-hole entrance, with some matrix being ablated, exposing the carbon fibers. Due to the different thermal conductivity of carbon fiber in the axial and radial directions, regions with different carbon fiber orientations exhibit varied features. As shown in Figure 9b,c, the region with carbon fibers aligned parallel to the radial direction of the micro-hole shows noticeable ablation at the fiber ends. Additionally, residual SiC matrix material can be observed on the carbon fibers, especially farther from the micro-hole. The wall of the micro-hole displays a zigzag-shaped morphology and broken carbon fibers, suggesting the effects of thermal expansion, as shown in Figure 9d. In contrast, carbon fibers aligned perpendicular to the radial direction of the micro-hole show clear sintering features, as seen in Figure 9e.

**Figure 10:**
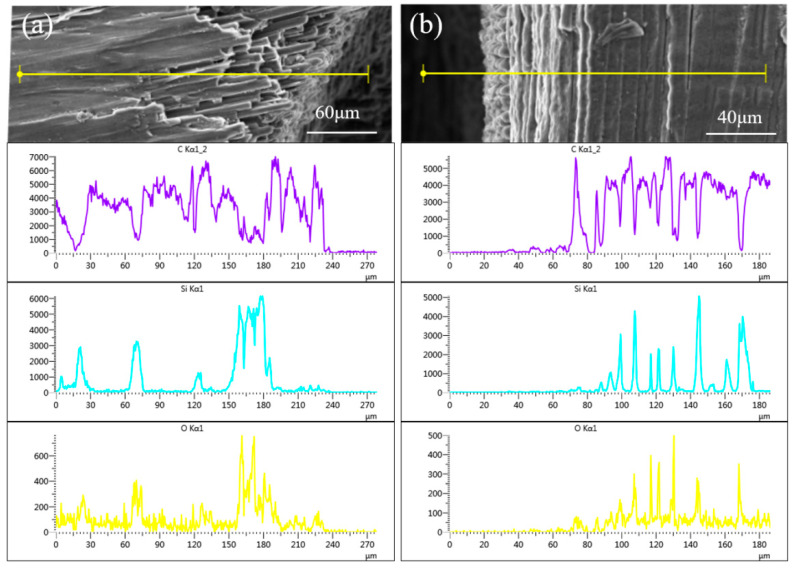
Elemental analysis on the adjacent regions of micro-holes entrance: (**a**) Linear elemental distribution of region with carbon fiber parallel to micro-hole radial direction, (**b**) Linear elemental distribution of region with carbon fiber perpendicular to micro-hole radial direction, where the purple represents carbon, the green represents silicon, and the yellow represents oxygen.

The EDS analyses on the regions with different carbon fiber orientations reveal the extent of oxidation in the superficial layer, as shown in Figure 10. Generally, the C exhibits the dense distribution, as it is contained in the fiber and matrix. Comparatively, the C distribution perpendicular to the carbon fiber axial direction is more uniform, while, at a certain angle, the C peak width is relatively wider. Compared with the C distribution, the difference in Si distribution is more pronounced, which should be attributed to the inhomogeneity of SiC in the microregion. The O distribution indicates that the oxidation has extended to an inner position. Based on the EDS results, the region with carbon fibers aligned with the radial direction of the micro-holes exhibits greater oxidation depth, which indicates a relatively worse interface; however, the inner oxidation is less than 100 micrometers, preventing deeper oxidation [27].

### 3.2. Comparison of Micro-Holes in C_f_/SiC Composite Processed by Laser Drilling in Air and with Waterjet

**Figure 11:**
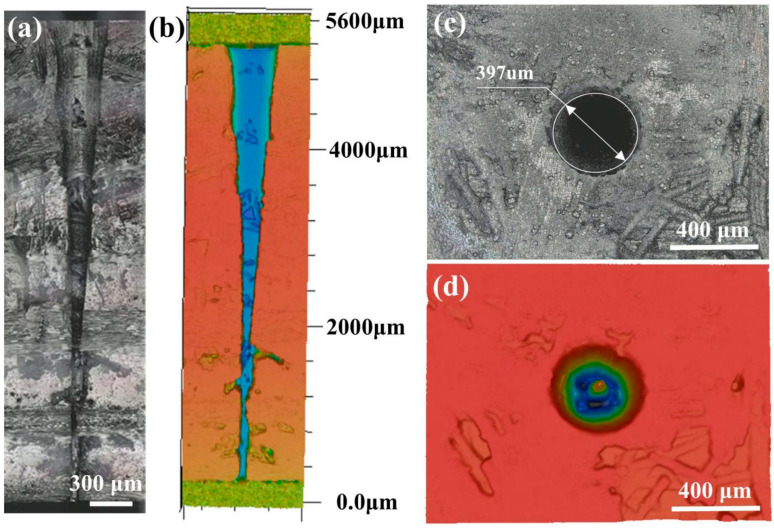
Typical morphology of micro-hole in the C_f_/SiC composite prepared by nanosecond laser drilling in air: (**a**) Cross-sectional shape, (**b**) Variation of dimension with depth, (**c**) Morphology of micro-hole entrance, (**d**) Shape of micro-holes.

To further verify the laser-drilling effect with different coupling media, the micro-holes with a diameter of about 400 µm were processed in the C_f_/SiC composite plate with a thickness of 5 mm. Similar laser parameters have been adopted for comparison, but the micro-holes were processed in air and waterjet successively. The waterjet velocity was set as 9.61 m/s. To realize the fabrication of micro-holes with a high aspect ratio in the C_f_/SiC composite, the focus compensation method was used with optimized parameters (see Table 3). As shown in Figure 2, the fabrication of micro-holes in the C_f_/SiC composite plate includes the initial nanosecond laser drilling in air and the subsequent reaming by coaxial waterjet-assisted nanosecond laser drilling. Due to the plasma, oxidation, spattering, and so on, the laser-drilled micro-holes exhibit diversified morphology. As shown in Figure 11, the micro-hole prepared by nanosecond laser drilling in air with the *Z*-axis compensation method had an entrance diameter of about 397 μm and an exit diameter of about 57 μm. The cross-sectional morphology shows that the micro-hole has a typical cone shape with a gradually decreasing diameter, as shown in Figure 11a,b. The micro-hole entrance exhibits a regular circular shape, but small-sized grooves could be observed on the inner wall, as shown in Figure 11c,d. Such a taper shape indicates it is difficult to realize ideal micro-holes with a high aspect ratio in the C_f_/SiC composite.

**Figure 12:**
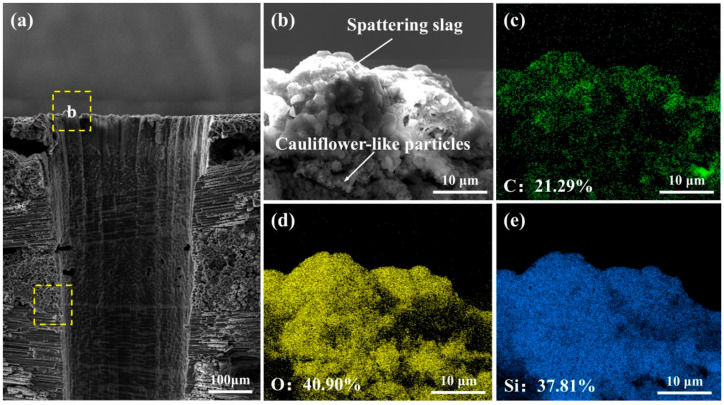
SEM observation and EDS analyses on the upper region of the micro-hole in C_f_/SiC composite processed by nanosecond laser drilling in air: (**a**) Microstructure of inner wall and interface, where the yellow frame at the bottom corresponds to the position shown in Figure 13, (**b**) Morphology of spattering slag on entrance, (**c**) Elemental distribution of C in spattering slag, (**d**) Elemental distribution of O in spattering slag, (**e**) Elemental distribution of Si in spattering slag (elemental content is atomic percent).

SEM observation on the micro-hole entrance shows the groove shape, which reflects the laser-drilling effect, as shown in Figure 12a. In addition, the deposits with small sizes could be observed, which should be the spattering slags. Further observations on the spattering slags exhibit they are mixtures of particles with various sizes, as shown in Figure 12b. The small particle clusters with cauliflower-like morphology could be found between the composite and spattering slags, which should be formed at the initial state. The EDS analyses on the spattering slags indicate they should be SiC and SiO_2_, as shown in Figure 12c,d. The analyzed spattering slags contain C, O, and Si elements with ratios of 21.29%, 40.90%, and 37.81%, respectively. Due to the deposition of the spattering slags, there is a high porosity inside, which also confirms the periodic feature [34]. During the laser processing, the high-temperature, high-pressure, and high-density plasma is formed above the ablated area, which generates shock waves and takes away some melts-forming deposited slags [39]. Because of the involvement of air, the vaporized C and Si react with the oxygen and form small particles of SiO_2_ and CO_2_ gas. The SiO_2_ particles deposited on composite surface result in the cauliflower-like deposits at the initial stage, while the periodic laser drilling leads to the layer structure and pores inside [40].

**Figure 13:**
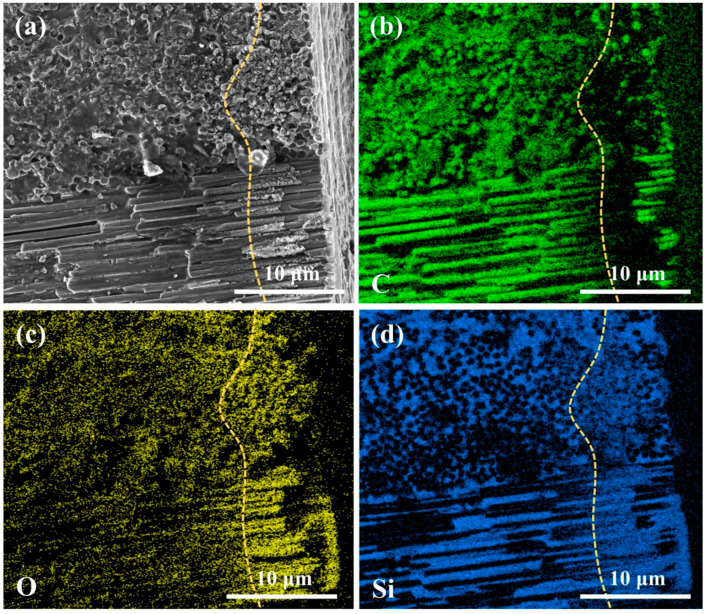
Typical SEM observation and corresponding EDS analyses on the cross-section adjacent to the middle region of micro-hole processed by nanosecond laser drilling in air: (**a**) Cross-sectional microstructure showing thermal effect, where the yellow line in the figure represents the boundary of the heat-affected zone, (**b**) Elemental distribution of C, (**c**) Elemental distribution of O, (**d**) Elemental distribution of C.

Further SEM observation and EDS analyses of the micro-hole wall surfaces at deeper positions demonstrate that the laser processing has caused internal oxidation in the C_f_/SiC composite. As shown in Figure 13a, the cross-sectional microstructure exhibits the small particles attached on the surface of carbon fibers. The depth of such morphology is about 5 micrometers and shows little variation across carbon fibers with different orientations. There seems to be, however, a slight difference in oxidation depth, which can be attributed to varying porosity in the local regions. EDS analyses indicate that the small particles are enriched with O and Si, as shown in Figure 13b–d. These results confirm that the small particles are likely SiO_2_. According to previous studies [30,41], the interface plays an important role in the oxidation of carbon fiber reinforced composites. For the present C_f_/SiC composite, the pore defects are mainly located in the SiC matrix or at the interface of carbon fibers. As a result, the oxidation occurs in the matrix interval, forming oxides along the interfaces of the carbon fibers. Therefore, it is necessary to isolate the laser-processed region from the air to reduce oxidation.

**Figure 14:**
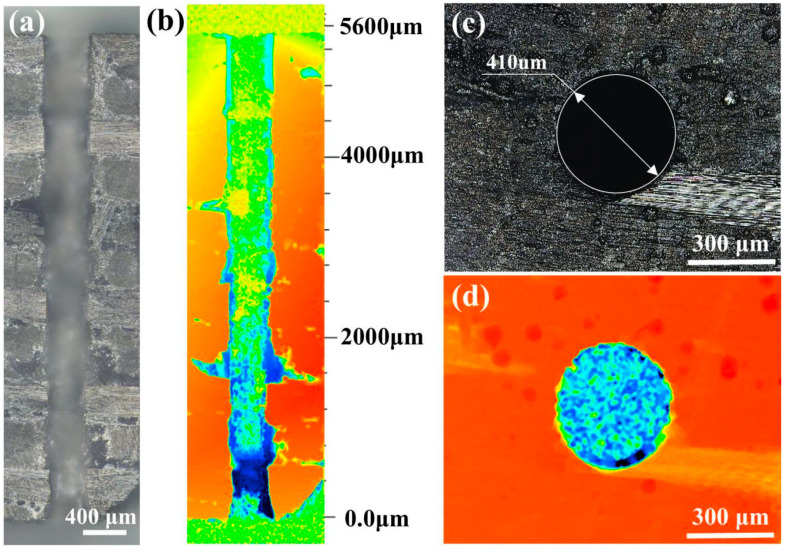
Typical morphology of coaxial waterjet-assisted nanosecond laser-drilled micro-hole in C_f_/SiC composite: (**a**) Cross-sectional shape, (**b**) Variation of dimension with depth, (**c**) Morphology of micro-hole entrance, (**d**) Shape of micro-hole.

The typical morphology and shape of the micro-holes subsequently processed by coaxial waterjet-assisted nanosecond laser drilling are shown in Figure 14. Compared to the micro-hole prepared by nanosecond laser drilling in air, the coaxial waterjet-assisted nanosecond laser-drilled micro-hole exhibits a small taper, as shown in Figure 14a. The relatively homogeneous diameter throughout the entire micro-hole indicates that the laser drilling is minimally affected by external factors, which should be attributed to the efficient removal of plasma and corresponding vaporized particles. Analysis of the cross-sectional structure of the micro-hole reveals that it still has the decreasing tendency in diameter from entrance to exit, as shown in Figure 14b. The extended pores of a relatively big size could be observed clearly along the layer interface, which should be ascribed to the local exfoliation caused by drastic temperature change. The micro-hole has an entrance diameter of 410 μm and an exit diameter of about 347 μm, as shown in Figure 14c,d. Comparatively, both the entrance and exit maintain a regular circle shape, implying relatively high drilling precision.

**Figure 15:**
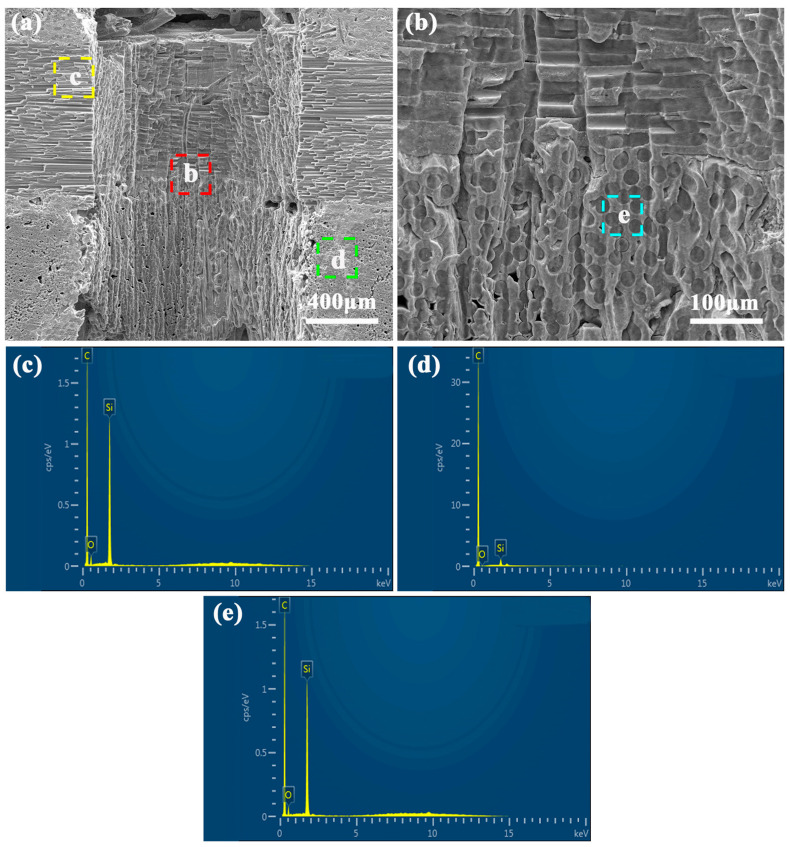
SEM observation and EDS analyses on the upper region of coaxial waterjet-assisted nanosecond laser-drilled micro-holes in the C_f_/SiC composite: (**a**) Morphology of inner wall and interface, (**b**) Surface morphology of laser-ablated composite, (**c**) EDS analysis on region adjacent to micro-hole wall with parallel carbon fibers, (**d**) EDS analysis on micro-hole wall, (**e**) EDS analysis on region adjacent to micro-hole wall with perpendicular carbon fibers.

The typical SEM observations and EDS analyses on the coaxial waterjet-assisted nanosecond laser-drilled micro-holes are shown in Figure 15. The results indicate that the waterjet significantly alters the surface morphology of the micro-holes. Relatively large pore defects are observed, and they tend to appear along the layer interface, as shown in Figure 15a. Moreover, the micro-hole wall exhibits a zigzag-like surface feature, indicating the erosion effect of the waterjet. The rapid heating from laser irradiation and the drastic cooling from the waterjet induce internal stress, resulting in cracks within the composite and the formation of small grooves in localized areas. Further observation of the layer interfaces, with carbon fibers oriented in different directions, reveals varied surface features, as shown in Figure 15b. The layer with parallel carbon fibers exhibits many cracked or exfoliated fibers, while the layer with perpendicular carbon fibers displays a more cracked matrix. Notably, the cross-sectional SEM images and micro-hole wall morphologies reveal no discernible microstructural changes indicative of a heat-affected zone (HAZ), which makes a sharp contrast to the significant microstructural alterations observed in Figure 13. Furthermore, EDS analyses show almost no detectable oxygen content in the processed regions, suggesting an absence of oxide layer formation, as shown in Figure 15c–e. This strongly implies that the waterjet environment effectively suppresses thermal accumulation at the composite surface by rapidly cooling the interaction zone and continuously removing molten material and plasma. Consequently, the formation of an HAZ is largely prevented during the waterjet-assisted laser drilling. EDS analyses on different regions reveal that the region adjacent to the micro-hole wall with parallel carbon fibers has a relatively high O content. Comparatively, the region adjacent to the micro-hole wall with perpendicular carbon fibers has a relatively low O content, while the micro-hole wall surface of the micro-hole exhibits oxygen content similar to that of the parallel carbon fibers. This indicates that the diffusion of oxygen along the carbon fibers is easier than that traversing the carbon fibers. Although a trade-off remains in place between micro-hole wall quality and HAZ control, this combined process markedly enhances the overall performance and structural controllability of micro-hole fabrication, thereby providing robust support for relevant applications.

**Figure 16:**
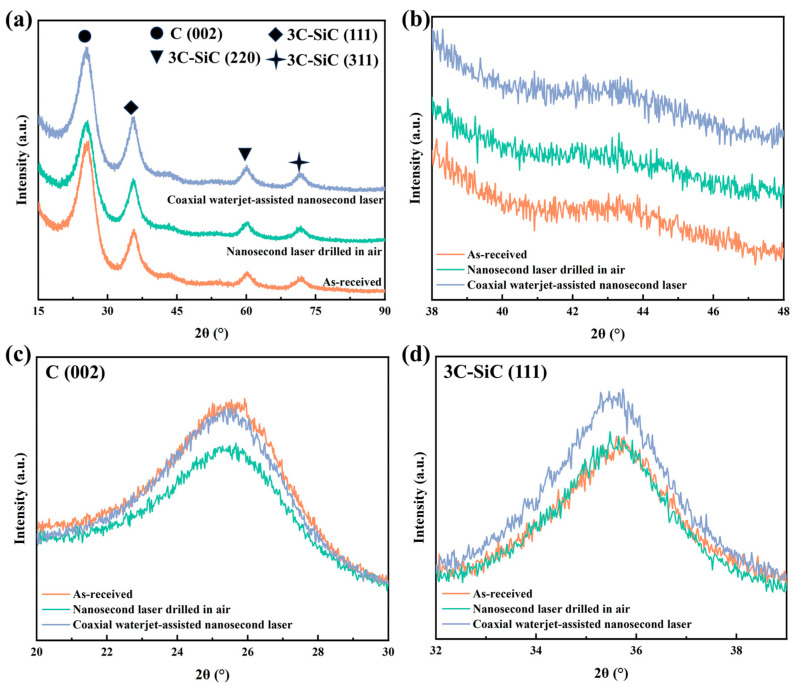
XRD patterns of C_f_/SiC composites with different states: (**a**) Full patterns of all specimens, (**b**) Enlarged XRD patterns between 40° and 46°, (**c**) Enlarged XRD patterns along 002_C_ diffraction pattern, (**d**) Enlarged XRD patterns along 111_SiC_ diffraction pattern.

To verify the effect of the coupling medium on laser-processed C_f_/SiC composites, XRD analysis was performed on their processed surfaces, comparing them to the original composite. As shown in Figure 16, the XRD patterns demonstrate similar diffraction peaks, but with different intensities. Clearly, all specimens are primarily composed of SiC and C, as shown in Figure 16a. According to PDF#01-073-1665, the diffraction peaks at 2θ of 35.7°, 60.1°, and 71.9° correspond to the crystallographic plane of (111), (220), and (311) for 3C-SiC, respectively. The diffraction peak at 2θ of 25.4° corresponds to the C phase, identified using PDF#00-034-0567. Further analyses of the XRD patterns also reveal special peaks between 40° and 47°, as shown in Figure 16b. The broad diffraction peaks suggest the formation of amorphous structure in the composites with different states, which is likely a mixture of SiO_2_ and Si [42]. Comparatively, the amorphous features in the C_f_/SiC composites processed by nanosecond laser drilling in air and coaxial waterjet-assisted nanosecond laser drilling are more obvious than those in the as-received C_f_/SiC composite. Detailed analysis of the diffraction peaks for 002_C_ demonstrates a relatively weakened intensity in the C_f_/SiC composites processed by nanosecond laser drilling in air and coaxial waterjet-assisted nanosecond laser drilling, as shown in Figure 16c. That should be partly ascribed to the ablation or cracking of carbon fibers during the laser processing. In contrast, analysis of the diffraction peaks for 111_SiC_ exhibits an increased intensity in the C_f_/SiC composites processed by coaxial waterjet-assisted nanosecond laser drilling, as shown in Figure 16d. Such results further confirm the chemical composition changes in the superficial layer during laser processing. During nanosecond laser drilling in air, the C_f_/SiC composite has contact with oxygen, generating a large number of SiC and SiO_2_ particles. These particles are sputtered onto the sidewalls, bottom, and surrounding areas of the micro-holes, forming a recast layer through counter-impact forces, which enhances the SiC diffraction peak.

**Figure 17:**
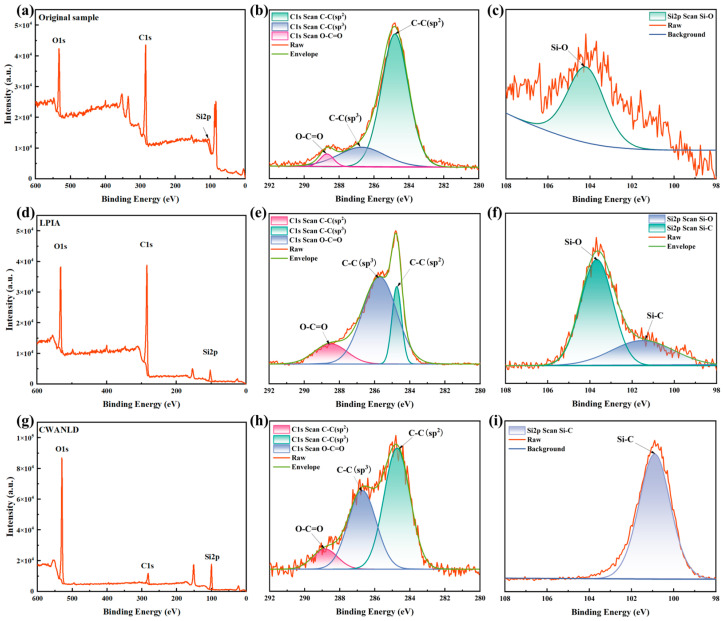
XPS analyses of C_f_/SiC composites with different states: (**a**) Full spectrum of as-received composite, (**b**) C1s spectrum of as-received composite, (**c**) Si2p spectrum of as-received composite, (**d**) Full spectrum of composite processed by nanosecond laser drilling in air, (**e**) C1s spectrum of composite processed by nanosecond laser drilling in air, (**f**) Si2p spectrum of composite processed by nanosecond laser drilling in air, (**g**) Full spectrum of coaxial waterjet-assisted nanosecond laser-drilled composite, (**h**) C1s spectrum of coaxial waterjet-assisted nanosecond laser-drilled composite, (**i**) Si2p spectrum of coaxial waterjet-assisted nanosecond laser-drilled composite.

X-ray photoelectron spectroscopy (XPS) was employed to analyze the elemental composition and chemical state of the surface layer in C_f_/SiC composites with different processing conditions. As shown in Figure 17a,d,g, the photoelectron peaks of C, Si, and O elements can be detected in the full spectra, although their positions differ slightly. For the as-received C_f_/SiC composite, the peaks corresponding to Si2p, C1s, and O1s are observed at 104.1 eV, 284.6 eV, and 532.6 eV, respectively. For the C_f_/SiC composite processed by nanosecond laser drilling in air, the peaks of Si2p, C1s, and O1s shift at 100.7 eV, 284.6 eV, and 530.0 eV, respectively. For the coaxial waterjet-assisted nanosecond laser-drilled C_f_/SiC composite, the peaks of Si2p, C1s, and O1s are observed at 102.8 eV, 284.6 eV, and 532.5 eV, respectively. The variation of Si2p and O1s peaks implies a reaction in the surface layer during laser processing. In general, the C1s spectrum is composed of photoelectron peaks corresponding to the chemical bonds of C–C(sp2), C–C(sp3), and O–C=O, while the Si2p spectrum reflects photoelectron peaks associated with Si–O and Si–C chemical bonds. For the as-received C_f_/SiC composite, the strongest peak in the C1s spectrum corresponds to C–C(sp2) bonds, while both the C–C(sp3) and O–C=O peaks are weak, as shown in Figure 17b. In comparison, the C_f_/SiC composite processed by nanosecond laser drilling in air exhibits the strongest C–C(sp3) peak, with a greatly weakened C–C(sp2) peak, as shown in Figure 17e. For the coaxial waterjet-assisted nanosecond laser-drilled C_f_/SiC composite, the C–C(sp2) peak becomes the strongest again and the C–C(sp3) peak weakens slightly, as shown in Figure 17h. Compared to the as-received C_f_/SiC composite, the O–C=O peak in laser-processed composite is enhanced, although it remains the weakest peak. These results imply the initial crystal structures have been partly destroyed and transformed into oxides, further confirming observations from the SEM analyses. In addition, the exfoliation of carbon fibers also contributes to such an evolution of C1s spectrum [33]. It is evident from the C1s spectrum that no C–Si bond is present, which can be attributed to the stripping of the SiC matrix and the oxidation of its residual components. The increased C–C(sp3) bond in the laser-processed C_f_/SiC composite further suggests the enhanced stability of the carbon fiber cross-section. Detailed analysis of the Si2p spectra of C_f_/SiC composites in different states reveals variations in the silicon oxide content. The as-received C_f_/SiC composite shows only Si–O bonds, indicating the presence of oxides in the surface layer, as shown in Figure 17c. For the C_f_/SiC composite processed by nanosecond laser drilling in air, the intensity of the Si–O bond increases, and a Si–C bond appears, as shown in Figure 17f. Interestingly, the coaxial waterjet-assisted nanosecond laser-drilled C_f_/SiC composite exhibits only the Si–C bond, as shown in Figure 17i, suggesting that the oxidation of the SiC matrix is effectively minimized.

### 3.3. Influence of Assisting Medium on Laser Ablation Behavior

According to previous research [43,44,45,46], the laser processing of C_f_/SiC composites mainly involves photothermal and photochemical transformation. Due to high-density laser energy and the photothermal effect, laser irradiation on a small region of the C_f_/SiC composite leads to a sharp increase of temperature, reaching the sublimation point, which causes the layer to separate through vaporization. Furthermore, the laser irradiation also induces bond-breaking and atomic transition, contributing to laser ablation and the formation of plasma [47]. For the C_f_/SiC composite, the average binding energies of the C–Si bond and C–C bond are 299 kJ/mol and 347 kJ/mol, corresponding to photon energies of 3.0 eV and 3.6 eV, respectively. The photon energy of the nanosecond laser, however, with a wavelength of 532 nm, is only 2.3 eV [48]. Therefore, multiphoton nonlinear absorption of the laser energy is required to break the chemical bonds in C_f_/SiC composite. The combined photothermal and photochemical effects lead to plasma formation above the irradiated region, which promotes oxidation in the presence of oxygen. The plasma, in combination with fine oxides, reflects the laser energy and influences laser ablation. Therefore, the assisting medium plays a crucial role in the laser processing of C_f_/SiC composite.

**Figure 18:**
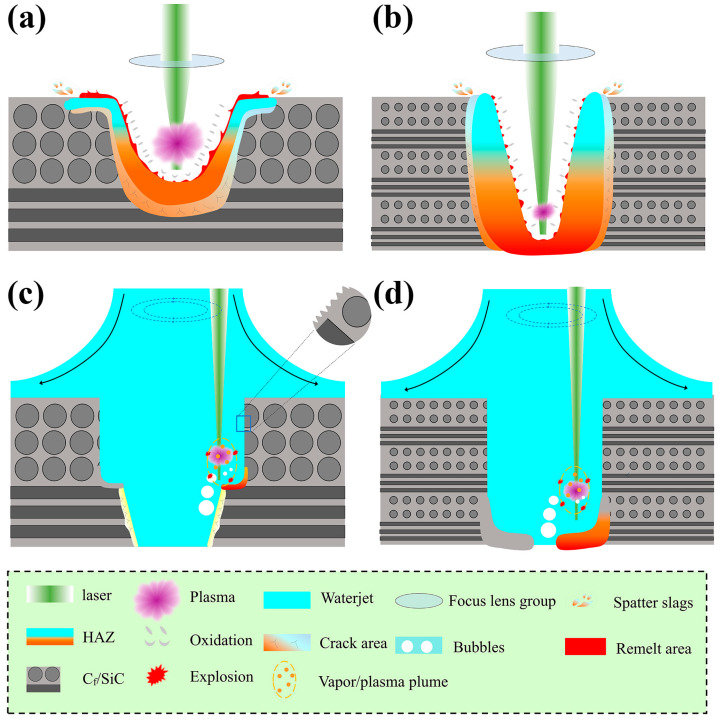
Schematic diagram of nanosecond laser drilling C_f_/SiC composites: (**a**) Initial stage and (**b**) final stage in air, (**c**) Initial stage and (**d**) final stage with coaxial waterjet.

In the present research, the influence of air and waterjet on the laser processing of C_f_/SiC composite is summarized in Figure 18. For the C_f_/SiC composite processed by nanosecond laser drilling in air, the presence of oxygen promotes oxidation and facilitates plasma formation. As shown in Figure 18a, laser irradiation on a small-size region leads to remelting and vaporization, generating smoke and ultrafine particles. The photochemical effect of the laser accelerates the ionization of these ultrafine particles, resulting in plasma formation. Simultaneously, a heat-affected zone (HAZ) is formed near the micro-holes, further promoting internal oxidation. The drastic temperature change results in localized micro-explosions, causing slag to be spattered around the entrance or onto the wall of the micro-hole. Due to the presence of oxygen, the spattered slags undergo rapid oxidation, forming a dense layer. The rapid cooling rate and the mixture of multiple phases facilitate the formation of an amorphous structure locally. The reflection of plasma on the laser beam, combined with the increased difficulty in expelling slag, gradually weakens the drilling effectiveness. This combination of plasma, energy dissipation, and slag spattering leads to a reduction in the laser energy available for drilling, as illustrated in Figure 18b. Therefore, the laser drilling of micro-holes in air results in a distinct taper shape. This characteristic limits the use of laser drilling in air for the fabrication of micro-holes in C_f_/SiC composites, particularly for high aspect-ratio micro-holes.

In contrast, the coaxial waterjet-assisted laser-drilled micro-holes in the C_f_/SiC composite significantly improves the quality. Benefiting from the initial micro-hole prepared by nanosecond laser drilling in air, the waterjet could flow through smoothly, effectively removing the remelted slag and maintaining the dimension of the micro-hole, as shown in Figure 18c. In addition, the waterjet largely eliminates the vaporized composite and inhibits the formation of plasma, thereby enhancing the absorption of laser energy in specific micro-regions and enabling efficient laser ablation. As a result, the micro-hole features clean wall surface with minimal oxides. The absence of accumulated slag on the micro-hole walls ensures that the laser processing follows the intended path, producing ideal micro-holes. Due to the better cooling effect of the waterjet, the HAZ could be well restrained in the laser-processed micro-holes with waterjet. The scouring effect of the waterjet and its rapid cooling can, however, cause the cracking of the carbon fibers, particularly those oriented perpendicular to the radial direction of the micro-hole. With the oxygen isolation provided by the waterjet, surface oxidation has been effectively suppressed, and almost no impurities or oxides are attached to the micro-hole walls. Even though vaporization and oxidation have been well restrained, laser irradiation still induces plasma and cavitation bubbles, which can affect the surface morphology to some extent [49,50,51]. It is worth noting that the growth and collapse of cavitation bubbles can create transient high-temperature and high-pressure “hotspots” in localized regions, leading to potential thermal stress and surface defects. To minimize these effects, the waterjet parameters were carefully optimized to maintain a high-speed laminar flow in the laser processing zone. This stable flow promotes the rapid removal of nascent bubbles and plasma, suppresses prolonged bubble retention and aggregation, and prevents the high-energy collapse of bubbles, which significantly reduces hotspot formation. As a result, thermal accumulation is minimized, and continuous improvement in the integrity and surface quality of the processed C_f_/SiC composites can be achieved, as shown in Figure 18d. In general, laser processing in air allows for the relatively rapid drilling of micro-holes in the C_f_/SiC composites with simple equipment requirements; however, the resulting shape and dimensions of the micro-holes are often unsatisfactory. Nevertheless, the pre-drilled micro-holes can facilitate subsequent coaxial waterjet-assisted laser drilling. The simultaneous cleaning and isolation effects of coaxial waterjet-assisted laser drilling effectively expand the pre-drilled micro-holes, improving their shape and dimension. Therefore, combining laser drilling in air and coaxial waterjet-assisted laser drilling enables the fabrication of high aspect-ratio micro-holes in C_f_/SiC composites by leveraging the advantages of both methods. Further optimization of these parameters could enable highly efficient micro-hole drilling in C_f_/SiC composites.

## 4. Conclusions

In the present study, the effect of waterjet velocity on the coaxial waterjet-assisted nanosecond laser drilling of micro-holes in the C_f_/SiC composite is investigated. Then, the optimized coaxial waterjet-assisted nanosecond laser drilling, combined with nanosecond laser drilling in air, is applied to machine micro-holes with a high aspect ratio in the C_f_/SiC composite. Surface morphology, reacted product, and micro-hole shape are studied thoroughly. The following conclusions can be drawn:

(1) For the coaxial waterjet-assisted nanosecond laser drilling of micro-holes in the C_f_/SiC composite, increasing the waterjet velocity enhances the material removal rate and micro-hole depth, while reducing the micro-hole diameter and taper angle. The coaxial waterjet isolates the laser-ablated region and rapidly cools the corresponding area, which results in the formation of a mixture of SiC, SiO, and Si on the surface. With the increasing of coaxial waterjet velocity, the morphology of residual surface products changes from a net-like structure to individual spheres. Coaxial waterjet-assisted laser drilling with a waterjet velocity of 9.61 m/s provides a well-balanced trade-off between efficiency and quality for micro-hole fabrication.
(2) The nanosecond laser drilling in air fabricates micro-holes in the C_f_/SiC composite with low equipment requirements, but the micro-holes have an obvious taper feature. The accumulation of spattered slag, plasma, and energy dissipation causes the micro-hole diameter to decrease progressively with increasing depth, which limits its application in creating micro-holes with high aspect ratios.
(3) The application of coaxial waterjet-assisted nanosecond laser drilling to a micro-hole prepared by nanosecond laser drilling in air could effectively expand its diameter. The fabricated micro-hole exhibits a very small taper angle, a clean wall surface, and minimal reaction products. Compared with the nanosecond laser drilling in air, the subsequent coaxial waterjet-assisted nanosecond laser drilling could fabricate micro-holes of superior quality in the C_f_/SiC composite. This technique, combining laser drilling in air with subsequent coaxial waterjet-assisted laser drilling, shows great potential for fabricating high-quality micro-holes with high aspect ratios in C_f_/SiC composites.

## Figures and Tables

**Table 1:** Physical properties of the used C_f_/SiC composite.

Diameter of Carbon Fiber (μm)	Volume Fraction of Carbon Fiber (%)	Density (g/cm^3^)	Open Porosity (%)	Closed Porosity (%)	Thickness of the Fiber Layer (μm)
8 ± 0.5	40% ± 2	1.9–2.0	6	15	258.5 ± 16.5

**Table 2:** Parameters of coaxial waterjet-assisted nanosecond laser drilling process for C_f_/SiC composites.

Parameter	Symbol	Value
Wavelength (nm)	λ	532
Pulse width (ns)	$t_{p}$	10
Repetition frequency (kHz)	f	30
Laser beam diameter (μm)	d	50
Focus length (mm)	$L_{f}$	167
Scanning spacing (mm)	$l_{1}$	0.02

**Table 3:** Optimized processing parameters for the laser drilling experiments.

Process Variables	Laser Frequency (kHz)	Waterjet Velocity (m/s)	Laser Defocus Distance (mm)	Spot Overlap Ratio (%)
Parameters	25	9.61	−1	55

## Data Availability

The original contributions presented in this study are included in the article. Further inquiries can be directed to the author.
